# Sox2 modulation increases naïve pluripotency plasticity

**DOI:** 10.1016/j.isci.2021.102153

**Published:** 2021-02-06

**Authors:** Kathryn C. Tremble, Giuliano G. Stirparo, Lawrence E. Bates, Katsiaryna Maskalenka, Hannah T. Stuart, Kenneth Jones, Amanda Andersson-Rolf, Aliaksandra Radzisheuskaya, Bon-Kyoung Koo, Paul Bertone, José C.R. Silva

**Affiliations:** 1 Wellcome Trust-Medical Research Council Cambridge Stem Cell Institute, University of Cambridge, Cambridge, UK; 2 Department of Biochemistry, University of Cambridge, Cambridge, UK; 3 Department of Genetics, University of Cambridge, Cambridge, UK; 4 Department of Medicine, Alpert Medical School, Brown University, Providence, RI, USA

**Keywords:** Biological Sciences, Cell Biology, Stem Cells Research

## Abstract

Induced pluripotency provides a tool to explore mechanisms underlying establishment, maintenance, and differentiation of naive pluripotent stem cells (nPSCs). Here, we report that self-renewal of nPSCs requires minimal Sox2 expression (Sox2-low). Sox2-low nPSCs do not show impaired neuroectoderm specification and differentiate efficiently *in vitro* into all embryonic germ lineages. Strikingly, upon the removal of self-renewing cues Sox2-low nPSCs differentiate into both embryonic and extraembryonic cell fates *in vitro* and *in vivo*. This differs from previous studies which only identified conditions that allowed cells to differentiate to one fate or the other. At the single-cell level self-renewing Sox2-low nPSCs exhibit a naive molecular signature. However, they display a nearer trophoblast identity than controls and decreased ability of Oct4 to bind naïve-associated regulatory sequences. In sum, this work defines wild-type levels of Sox2 as a restrictor of developmental potential and suggests perturbation of naive network as a mechanism to increase cell plasticity.

## Highlights

• Low Sox2 expression is sufficient for naive pluripotent stem cell self-renewal
• Low Sox2 expression does not impair neurectoderm differentiation *in vitro*
• Low Sox2 expression impairs Oct4 genomic occupancy
• Low Sox2 increases naive pluripotent stem cell plasticity *in vitro* and *in vivo*

## Graphical abstract

**Figure:**
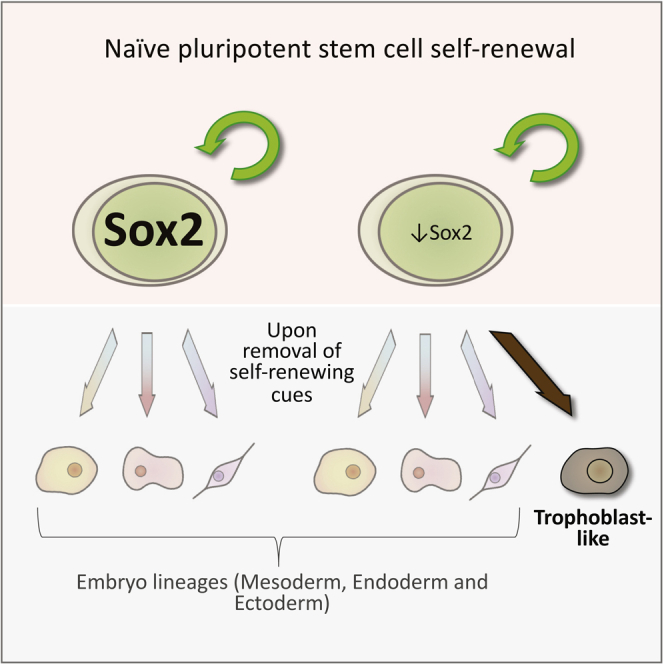

## Introduction

The naive epiblast is pluripotent as it has the potential to differentiate into any cell type of the embryo proper but cannot form extraembryonic lineages. Naive pluripotent stem cells (nPSCs) can be captured *in vitro* from the epiblast in the form of embryonic stem cells (ESCs) (Evans and Kaufman, 1981; Martin, 1981) and through reprogramming of differentiated cells in the form of induced pluripotent stem cells (iPSCs) (Takahashi and Yamanaka, 2006). ESCs and iPSCs are therefore excellent model systems to study the molecular mechanisms underlying pluripotency. Recently it has been demonstrated that treatment of ESCs with specific small molecules induces expanded differentiation potential (Yang et al., 2017a, 2017b). These cells could contribute to both trophectoderm and inner cell mass (ICM) in blastocyst chimaeras. However, the mechanism underlying this has not been elucidated.

Sox2 is a member of the SRY-related HMG-box family of transcription factors (Wright et al., 1993) and is a core pluripotency factor. Sox2 knockout in embryos results in peri-implantation lethality and its deletion in ESCs results in loss of self-renewal with the cells becoming trophoblast-like stem cells (Avilion et al., 2003; Masui et al., 2007). Sox2 was originally discovered as a putative DNA-binding partner of the central pluripotency factor Oct4 (Chew et al., 2005; Rodda et al., 2005; Yuan et al., 1995). However, self-renewing *Sox2*^*−/−*^ ESCs were generated in the presence of constitutive Oct4 expression, suggesting that the main role of Sox2 is to maintain Oct4 expression (Masui et al., 2007). Additionally, overexpression of Sox2 results in differentiation of ESCs (Kopp et al., 2008). It has been hypothesized that Sox2 acts as a neurectoderm specifier, which needs to be in balance with mesendoderm specifiers to result in pluripotency (Loh and Lim, 2011; Thomson et al., 2011). Despite these studies, the role and requirement of Sox2 in naive pluripotency remains unclear.

Here we utilized the process of induced pluripotency to investigate the biological role of Sox2. This uncovered the surprising role of Sox2 in restricting the potency of nPSCs to embryonic lineages only. This impacts on both the understanding of the role of core naive pluripotency factors and on the molecular basis governing the potency of nPSCs.

## Results

### Low Sox2 expression is compatible with nPSC self-renewal

**Figure 1:**
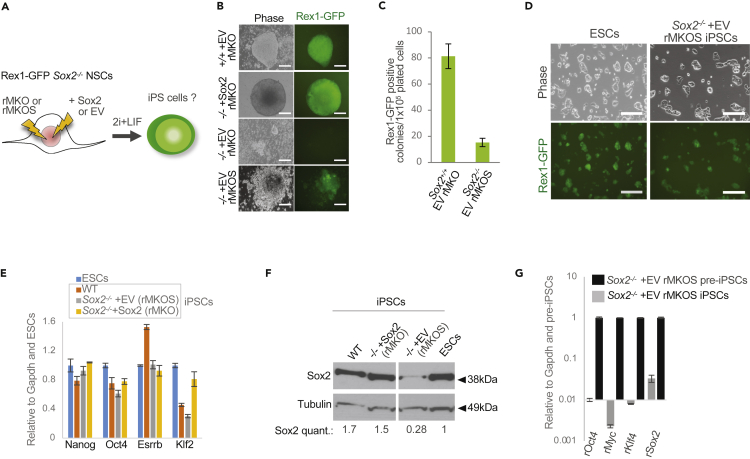
Generation of self-renewing iPSCs expressing low levels of Sox2 (A) Diagram of experimental design. rMKO and rMKOS indicate use of retroviral (r) vectors containing reprogramming cMyc (M), Klf4 (K), Oct4 (O), and Sox2 (S) transgenes. + EV (empty vector) or + Sox2 represent use of a PiggyBac plasmid containing a CAG promoter driving constitutive expression of either an empty or Sox2 transgene, respectively. (B) Phase and GFP images of emerging Rex1-GFP + iPSC colonies (n = 3). (C) Rex1-GFP + colony counts for indicated genotypes (n = 3). (D) Phase and GFP images of Sox2^−/−^ rMKOS iPSCs in 2iLIF post-selection. Rex1-GFP ESCs are shown as control. (E) qRT-PCR analysis for indicated pluripotency associated factors in iPSC and control ESC lines. (F) Western blot of Sox2 (≈40kDa) and Tubulin (≈50kDa) in iPSC and control ESC lines in 2iLIF with Sox2 quantification relative to ESCs, normalized to tubulin. Gap in Western blot represents removal of a non-relevant lane and image corresponds to same film exposure. (G) qRT-PCR of retroviral Sox2, Oct4, Klf4, and cMyc expression in Sox2^−/−^ rMKOS iPSCs relative to preiPSCs. Scale bars = 200μm. Error bars indicate standard deviation of replicate qPCR reactions (n = 3).

To investigate the role of Sox2 in the process of induced pluripotency, we used neural stem cells (NSCs) as a source of somatic donor material. To attempt the generation of *Sox2*^*−/−*^ iPSCs, we made initially *Sox2*^*−/−*^ NSCs using CRISPR/Cas9 (Figures S1A–S1D). Clonal lines were confirmed to have a frameshift deletion in the Sox2 codon, resulting in loss of Sox2 protein (Figures S1A and S1B). The *Sox2*^*−/−*^ NSCs maintained NSC morphology, proliferative ability, and expression of NSC markers (Figures S1C and S1D). The *Sox2*^*−/−*^ NSCs contained GFP and the blasticidin resistance genes under the endogenous Rex1 regulatory sequences to allow identification and selection for nPSC identity (Wray et al., 2011). *Sox2*^*−/−*^ NSCs were induced to reprogram by combining four or three of the classic Yamanaka retroviral factors: cMyc, Klf4, Oct4 and Sox2 (rMKOS) or cMyc, Klf4, and Oct4 (rMKO) (Takahashi and Yamanaka, 2006) (Figure 1A). Retroviral promoters are active in somatic cells but become epigenetically silenced in naive pluripotency (Hotta and Ellis, 2008). Therefore, we hypothesized that the retroviral Sox2 would drive reprogramming but become silenced after stabilization of the network. We also used defined culture conditions containing inhibitors of Mek/Erk and Gsk3b signaling (2i) supplemented with Leukaemia inhibitory factor (LIF) for optimal reprogramming efficiency (Silva et al., 2008). *Sox2*^*−/−*^ NSCs were able to reprogram upon addition of a constitutive exogenous Sox2 transgene (Figure 1B). In the absence of retroviral Sox2, *Sox2*^*−/−*^ NSCs failed to upregulate the Rex1-GFP reporter (Figure 1B). Surprisingly, multiple *Sox2*^*−/−*^ Rex1-GFP + colony-like groups of cells emerged when using rMKOS (Figure 1B). Independent experiments showed this to be 5 times less efficient compared to the reprogramming of WT NSCs (Figure 1C). Reprogrammed Sox2^−/−^ rMKOS iPSCs were passagable in 2iLIF culture conditions and expressed naive pluripotency markers (Figures 1D and 1E). Surprisingly, they also expressed Sox2 protein at very low levels (Figure 1F), which was due to a failure to fully silence retroviral Sox2 (Figure 1G). Importantly, this data indicates a strong selective pressure for cells expressing a minimal level of Sox2 protein and suggests that low Sox2 expression is compatible with maintenance of a nPSC molecular identity. Hereafter, we will refer to these *Sox2*^*−/−*^ rMKOS iPSCs as Sox2-low iPSCs for simplicity.

### Sox2-low iPSCs differentiate in serum plus LIF self-renewing conditions

**Figure 2:**
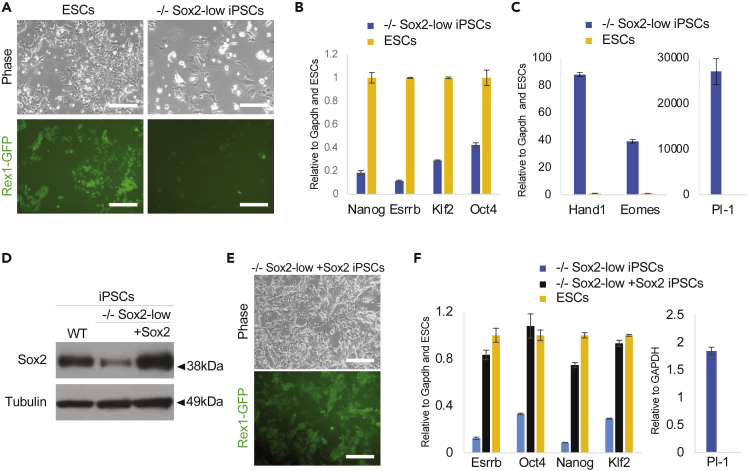
Sox2-low iPSCs do not self-renew in serum plus LIF (A) Phase and Rex1-GFP images of *Sox2*^*−/−*^ rMKOS (−/− Sox2-low) iPSCs and ESCs at passage 0 after medium switch from 2i plus LIF into serum plus LIF. (B and C) qRT-PCR analysis of pluripotency markers (B) and trophoblast markers (C) 3 days after switching into serum plus LIF medium. (D) Western blot of Sox2-low iPSCs in 2i plus LIF with and without rescue Sox2 transgene (+Sox2). Rex1-GFP WT iPSCs were used as control for Sox2 protein levels. (E) Phase and GFP images of −/− Sox2-low + Sox2 rescue iPSCs after 5 passages in serum plus LIF. (F) qRT-PCR analysis, after 3 days in serum plus LIF, of pluripotency factors and Pl-1 in −/− Sox2-low iPSCs with and without a rescue Sox2 transgene. ESCs were provided as control in the qRT-PCR analysis of pluripotency factors. Scale bars = 200μm. Error bars indicate standard deviation of replicate qPCR reactions (n = 3).

It has previously been reported that Sox2 repression in ESCs results in loss of pluripotency and differentiation toward the trophoblast lineage in serum plus LIF (SLIF) conditions (Masui et al., 2007). Therefore, we attempted to culture Sox2-low iPSCs in SLIF. Strikingly, Sox2-low iPSCs downregulated the Rex1-GFP reporter, and some cells gained a trophoblast-like morphology (Figure 2A). They also downregulated pluripotency marker expression and upregulated trophoblast markers (Figures 2B and 2C). In addition, cells were not passageable, demonstrating loss of self-renewal. To ensure that the differentiation phenotype was due to low Sox2 expression, we generated Sox2-low rescue iPSCs by transfecting these with a constitutive Sox2 transgene in 2iLIF (Figure 2D). Upon SLIF medium switch, and in contrast to Sox2-low iPSCs, rescue iPSCs self-renewed, maintained naive pluripotent gene expression and did not upregulate trophoblast marker expression (Figures 2E and 2F).

These results suggest that under weaker self-renewing culture conditions low levels of Sox2 are not sufficient to sustain a naive pluripotent identity. It also showed that at least a proportion of the cells differentiate toward the trophoblast lineage.

### Reduced Sox2 expression is associated with increased plasticity *in vitro*

**Figure 3:**
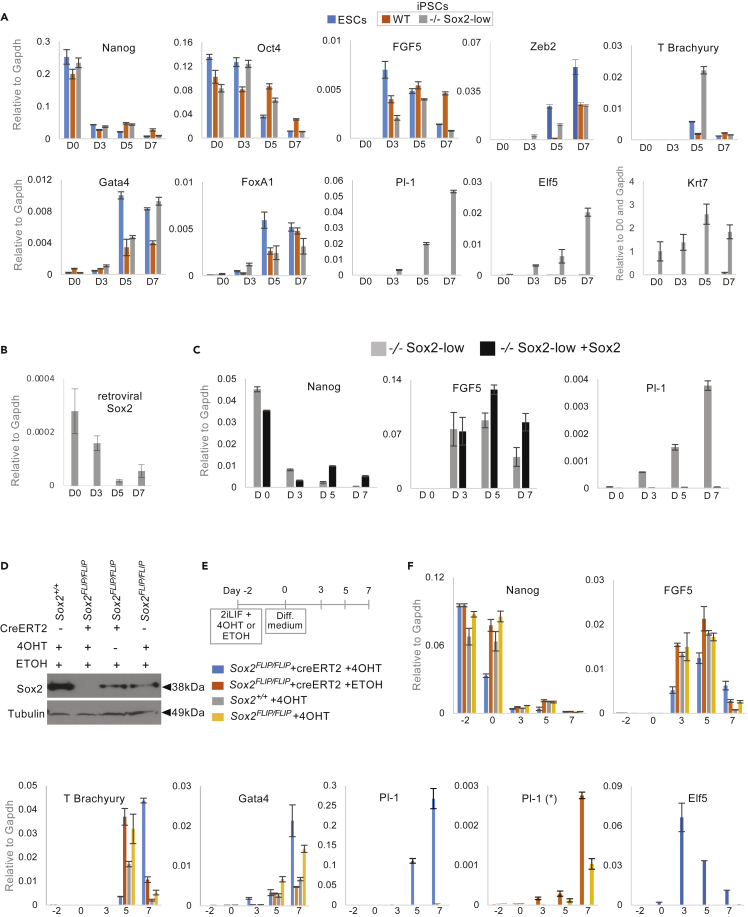
Sox2-low and null nPSCs differentiate into extraembryonic and all embryonic lineages (A) qRT-PCR analysis of embryoid body assay using *Sox2*^*−/−*^ (−/−) Sox2-low iPSCs, control iPSCs (WT) and ESCs showing RNA expression of markers of pluripotency (Nanog, Oct4), ectoderm (FGF5), mesoderm (T Brachyury and Zeb2), endoderm (FoxA1 and Gatat4) and trophoblast (Pl-1 and Elf5). (B) qRT-PCR analysis of embryoid body assay for retroviral Sox2 expression in −/− Sox2-low iPSCs. (C) qRT-PCR analysis of embryoid body assays of −/− Sox2-low and rescue (+Sox2) iPSCs, for expression of pluripotency (Nanog), late epiblast (FGF5) and trophectoderm (PL-1) markers. (D) Western blot showing Sox2 protein post tamoxifen (4OHT) treatment in *Sox2*^*FLIP/FLIP*^ ESCs with or without a constitutive CreERT2 transgene. Carrier only (ethanol, ETOH) and *Sox2*^*+/+*^ ESCs were used as additional controls. (E) Experimental design of embryoid body assay and of Sox2 deletion in *Sox2*^*FLIP/FLIP*^ ESCs. (F) qRT-PCR analysis of embryoid body assay using Sox2^FLIP/FLIP^ ESCs showing expression of pluripotency markers (Nanog), late epiblast (FGF5), mesoderm (T Brachyury), endoderm (Gata4), trophoblast (Pl-1 and Elf5). Pl1 (∗) chart omits *Sox2*^*FLIP/FLIP*^ CreERT2 ESC sample treated with 4OHT. Error bars indicate standard deviation of replicate qPCR reactions (n = 3).

To functionally characterize the potency of Sox2-low iPSCs we investigated their differentiation capacity in embryoid body (EB) assays. These efficiently downregulated pluripotency genes and upregulated both endoderm and mesoderm differentiation markers. Importantly, retroviral Sox2 was not upregulated during differentiation (Figure 3B). Because of the observed upregulation of trophoblast markers in Sox2-low iPSCs in SLIF, we also explored the expression of these. In contrast to control lines, Sox2-low iPSCs upregulated expression of trophoblast markers during EB differentiation (Figure 3A). Interestingly, and in contrast to Elf5 and Cdx2, the trophoblast marker Krt7 was already expressed prior to cell differentiation. This phenotype was fully rescuable upon the restoration of Sox2 protein levels by the transfection of a constitutive Sox2 transgene (Figures 2D and 3C). These results suggest that Sox2-low iPSCs have increased plasticity as they can differentiate into both embryonic and extraembryonic lineages.

Next we went on to test our findings in an independent nPSC system (Figures 3D–3F and S2A–S2C). *Sox2*^*FLIP/FLIP*^ ESCs have previously been generated, which delete endogenous Sox2 expression upon exposure to Cre (Figure S2A) (Andersson-Rolf et al., 2017). Notably, *Sox2*^*FLIP/FLIP*^ ESCs express Sox2 protein at a reduced level compared to WT ESCs (Figure 3D). This is likely the result of the inserted cassette sequences interfering with the fine regulation of endogenous Sox2. To permit inducible Sox2 deletion, *Sox2*^*FLIP/FLIP*^ ESCs were constitutively transfected with creERT2. These were then subjected to a tamoxifen time course to establish the earliest time point at which Sox2 protein is absent. By 36 hr Sox2 protein was undetectable, whereas Nanog and Oct4 were still expressed at high levels at 48 hr, suggesting that differentiation had not occurred (Figures S2B and S2C). Therefore, to investigate the potency of Sox2-null ESCs, we treated CreERT2 *Sox2*^*FLIP/FLIP*^ ESCs with tamoxifen (4OHT) for 48 hr and then performed EB differentiation (Figures 3D–3F). The absence of Sox2 protein did not affect the expected kinetics of downregulation of pluripotency markers or the upregulation of differentiation markers of all 3 germ layers (Figure 3E). Importantly, absence of Sox2 expression was also associated with a striking upregulation of trophoblast markers Pl-1 and Elf5 (Figure 3F). Interestingly, parental *Sox2*^*FLIP/FLIP*^ ESCs, which exhibit lower Sox2 protein expression compared to control ESCs, also exhibit significant upregulation of the trophoblast marker Pl-1 compared to control ESCs (Figure 3F).

Importantly, these results confirm that reduced Sox2 expression in nPSCs (iPSCs and ESCs) is associated with a gain in cell plasticity, that is, an ability to differentiate toward the extraembryonic trophoblast lineage in addition to the embryonic lineages.

### Low Sox2 expression does not impair neurectoderm differentiation

**Figure 4:**
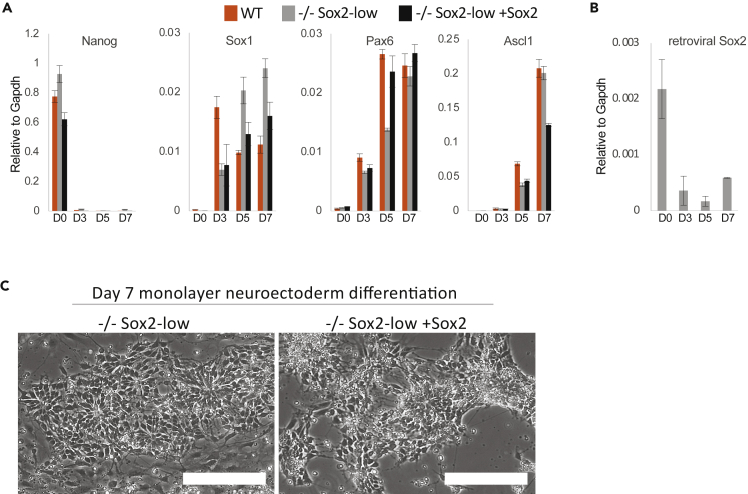
Low Sox2 expression is compatible with robust neuroectoderm differentiation (A) qRT-PCR analysis of *Sox2*^*−/−*^ (−/−) Sox2-low, −/− Sox2-low rescue (+Sox2) and WT iPSCs in a neurectoderm monolayer differentiation assay for markers of pluripotency (Nanog) and neurectoderm (Pax6, Ascl1, and Sox1). (B) qRT-PCR analysis of retroviral Sox2 in −/− Sox2-low iPSCs during neurectoderm monolayer differentiation. (C) Phase images of −/− Sox2-low iPSCs and Sox2-low rescue iPSCs on day 7 of the neurectoderm monolayer differentiation assay. Error bars indicate standard deviation of replicate qPCR reactions (n = 3).

Sox2 is thought to be required to drive ectoderm differentiation in pluripotent cells (Thomson et al., 2011). To examine the neurectoderm differentiation potential of these cells, we performed a neural monolayer differentiation (Ying et al., 2003). The Sox2-low iPSCs upregulated neural markers Sox1, Pax6, and Ascl1 to a similar level to WT iPSCs and Sox2-low rescue iPSCs and gained the characteristic neural rosette morphology, demonstrating efficient neural differentiation (Figures 4A and 4C). This also occurred without an increase of retroviral Sox2 expression (Figure 4B). These results suggest that reduced Sox2 expression does not impair robust neural differentiation *in vitro*.

### Reduced Sox2 expression is associated with increased plasticity *in vivo*

**Figure 5:**
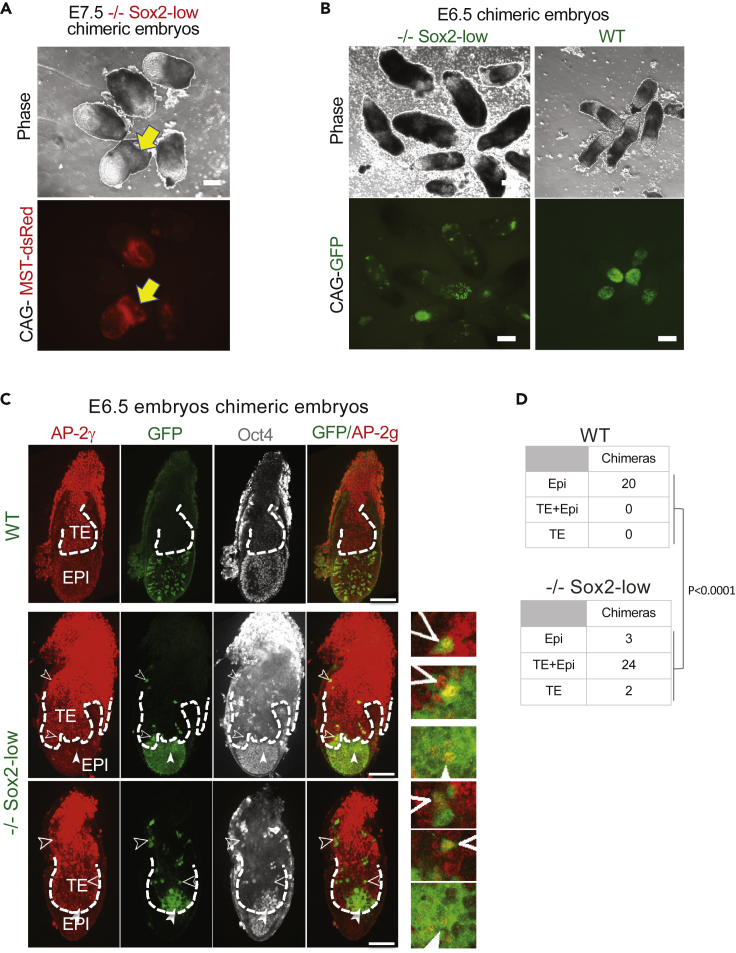
Sox2-low iPSCs exhibit increased plasticity *in vivo* (A) Phase and red fluorescence images of E7.5 chimaeras of *Sox2*^*−/−*^ (−/−) Sox2-low iPSCs expressing constitutively MST-dsRed (red fluorescence). Arrow indicates presumptive contribution to extraembryonic lineage(s). A non-chimeric embryo is included above the chimeric embryo with the yellow arrow (toward the left) to act as a negative control. Scale bars = 200μm. (B) Phase and GFP images of E6.5 chimeric embryos generated with either −/− Sox2-low or *WT* iPSCs constitutively expressing a GFP transgene. Please note that apparent difference in size is due to −/−Sox2 chimeric embryos having been imaged with some maternal tissue still attached. Scale bars = 200μm. (C) Single confocal microscopy sections of indicated genotype chimeric E6.5 embryos stained with trophoblast (AP-2γ) and epiblast (Oct4) markers. Filled arrowheads indicate examples of chimeric cells co-expressing Oct4 and AP-2γ markers. Non-filled arrowheads indicate examples of chimeric cells expressing AP-2γ only. Epiblast (EPI) and Trophoblast/trophectoderm (TE) embryo domains are separated by dashed line. Scale bars = 100μm. (D) Table showing compartmental contribution of *WT* or −/− Sox2-low iPSCs constitutively expressing a GFP transgene. Values in tables represent number of embryos. Fisher's exact test statistical analysis was used to calculate the significance of the difference in the proportion of embryos exhibiting trophectoderm contribution in the two groups. This was performed using GraphPad Prism software.

To investigate the effect of reduced Sox2 expression in differentiation *in vivo*, we performed morula aggregations with Sox2-low iPSCs. To visualize embryo chimerism we constitutively transfected Sox2-low iPSCs with MST-dsRed fluorescent protein. Contribution of Sox2-low iPS-derived cells could be seen in both epiblast and presumptive extraembryonic compartments at E7.5 (Figure 5A). In order to determine extraembryonic chimerism, we also generated Sox2-low and control iPSCs expressing constitutive GFP. As expected, WT iPSCs contributed exclusively to the E6.5 epiblast (Figures 5B–5D). In contrast, Sox2-low iPS-derived cells were found in both the epiblast and trophoblast, as defined by the AP2γ protein domain, compartments of the embryos (Figures 5B, 5C, 5E, and S3A). Lineage marker staining showed that 83% of Sox2-low chimaeras exhibit contribution to both the trophoblast and epiblast compartments (Figure 5D). In fact, 90% of all Sox2-low chimeras showed trophoblast contribution. Interestingly, some Sox2-low iPS-derived cells exhibited co-expression of trophoblast marker AP-2γ and epiblast marker Oct4 (filled arrowheads, Figure 5C) and this occurred in both the epiblast and trophoblast embryo compartments suggesting that this is an intermediate stage of cells changing from a pluripotent epiblast into a trophoblast identity.

To define when Sox2-low iPS-derived cells start contributing to the trophoblast lineage, we injected these at the morula stage and assessed chimerism at the blastocyst stage. Interestingly, almost all of the chimaeras showed contribution to the epiblast only (Figures S3B and S3C). This data support *in vitro* assays showing that Sox2-low iPSCs only acquire trophoblast lineage differentiation potential when in an environment no longer supportive of naive pluripotent cell identity.

Overall, these data demonstrate that Sox2-low iPSCs are competent to contribute to both embryonic and extraembryonic embryo development.

### Sox2-low iPSCs have weaker epiblast identity

To ascertain the identity of Sox2-low iPSCs we performed single-cell RNA-seq on these and control WT and Sox2-low + Sox2 rescue iPSCs. Principle component analysis showed that Sox2-low iPSCs can be separated from control lines (Figure S4A). Importantly, the rescue line clustered together with the parental line clearly indicating that the Sox2 expression level is the reason for grouping cells.

**Figure 6:**
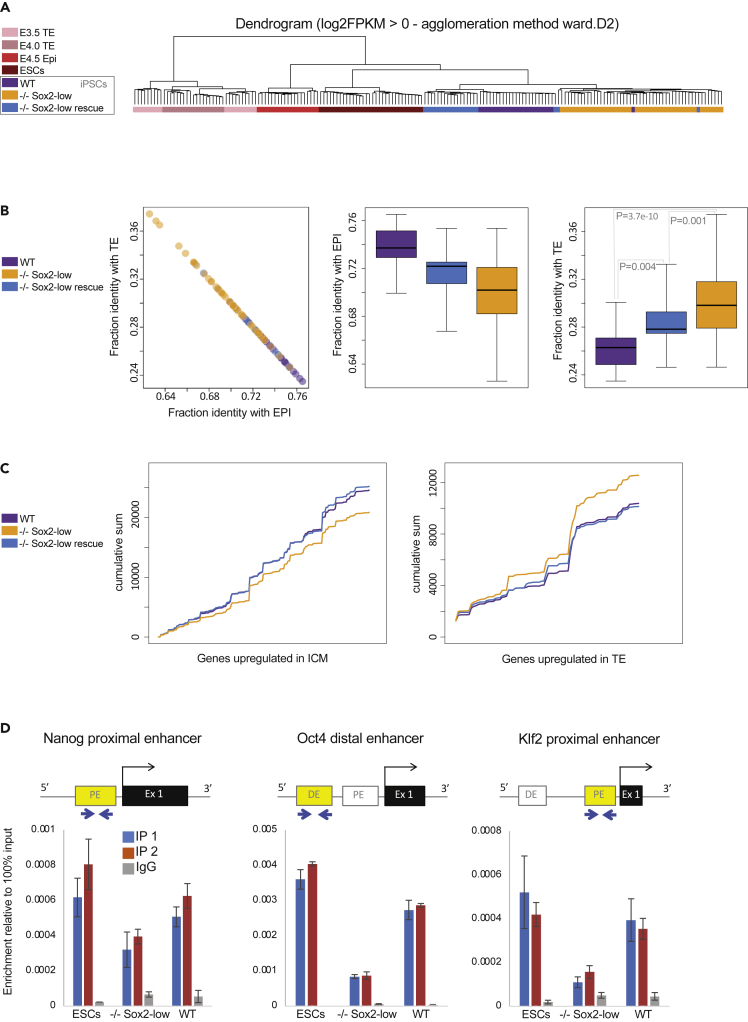
Sox2-low iPSCs have weaker epiblast identity (A) Dendrogram computed with the top variable genes among the selected cell types (FPKM >1, logCV2 > 0.5, n = 1,446). Trophectoderm/trophoblast (TE) E3.5 and E4.0 Single-cell RNA-sequencing *(*scRNA-seq) data is from (Deng et al., 2014), E4.5 Epiblast (Epi) scRNA-seq data is from (Mohammed et al., 2017), scRNA-seq ESC data is from (Sousa et al., 2018). (B) Scatterplot of fraction of identity between WT, −/− Sox2-low, −/− Sox2-low rescue iPSC line samples and embryo lineages (Epi and TE). Boxplot of the distribution of the fraction of identity between each population. Student's t-test was applied to calculate the significance of the differences in similarity to TE identity between −/−Sox2-low, WT and −/−Sox2-low rescue iPSCs. (C) Cumulative sum for genes upregulated in TE or ICM (Blakeley et al., 2015), computed with gene expression value for WT, −/− Sox2-low, −/− Sox2-low rescue iPSC line samples. (D) Oct4 chromatin immunoprecipitation in Rex1-GFP ESCs and iPSCs in 2iLIF. IP = immunoprecipitation; IgG = negative control. Error bars represent the standard deviation of three technical qPCR replicates. IP1 and IP2 represent independent Oct4 immunoprecipitations. IgG represents negative control normal IgG immunoprecipitation.

Gene ontology enrichment for genes differentially expressed between Sox2-low iPSCs and control iPSCs revealed only 325 differentially expressed genes between the different genotypes with no strongly enriched GO terms (Figures S4B and Data S1). In addition, no Sox family member gene was found upregulated in Sox2-low iPSCs, eliminating that way possible functional redundancy (Corsinotti et al., 2017). In agreement with Sox2-low iPSCs having a naive identity they clustered closely with control iPSCs, ESCs and E4.5 naive epiblast cells and separate from embryo trophoblast/trophectoderm (TE) cells (Figure 6A). However, when looking at the identity of single iPSCs on a continuum from an embryonic TE or naive epiblast perspective Sox2-low iPSCs were generally found in the fractions further away from the epiblast and nearer to the TE molecular identities relative to control iPSCs (Figure 6B). We also looked at accumulative gene expression for genes known to be associated with either the TE or with the ICM of embryos (Blakeley et al., 2015) (Figure 6C). Again, this revealed that Sox2-low iPSCs can be distinguished from WT and rescue lines as they display lower ICM and higher TE gene expression accumulation relative to control iPSCs.

ScRNA-seq analysis suggest that observed increased plasticity of Sox2-low cells is a functional property present at the single-cell level, as opposed to cell population heterogeneity.

### Reduced Sox2 expression impairs Oct4 DNA binding

Sox2 is thought to be a DNA-binding partner to Oct4, and Oct4 inhibits extraembryonic differentiation (Niwa et al., 2000; Reményi et al., 2003). Therefore, we hypothesized that reduced Sox2 protein level may impact directly or indirectly on Oct4 DNA-binding ability in nPSC. Consistent with this notion, Oct4 chromatin immunoprecipitation showed reduced Oct4 genomic occupancy in Sox2-low iPSCs at key naive pluripotent regulatory sequences (Figure 6D).

These results suggest that reduced Oct4 occupancy at naive associated regulatory sequences combined with closer TE molecular identity relative to controls are likely underlying causes rendering Sox2-low iPSCs competent to also contribute to the trophoblast lineage.

## Discussion

Here we show that reduced levels of Sox2 are compatible with self-renewal of nPSCs in 2iL conditions, making these a good model to study the role of Sox2 in early development.

These cells differentiate in SLIF demonstrating fragility in their pluripotent network. This fragility is likely due to the basal TE molecular identity and to the reduced Oct4 DNA-binding at naïve-associated regulatory sequences. Importantly, not only these cells can contribute toward the neural fate but also show increased cell plasticity, that is, they can differentiate into both embryonic and extraembryonic cell fates. This highlights a mechanism, modulation of Sox2 expression to lower than 30% of WT levels, by which a nPSC is competent, upon differentiation signals, to contribute toward both embryonic and extraembryonic cell fates; previous studies only identified conditions that allowed cells to differentiate to one fate or the other (Masui et al., 2007; Nichols et al., 1998).

Sox2 and Oct4 have been hypothesized to bind together at Oct/Sox elements to drive the pluripotency network (Chew et al., 2005; Rodda et al., 2005). It has been shown that Sox2 binds to the DNA target sequence first and that it recruits Oct4 (Chen et al., 2014). However, Oct4 and Sox2 were also reported to operate in a largely independent manner and their impact on each others ability to bind to regulatory sequences may be indirect and regulated by chromatin accessibility (Friman et al., 2019). Independent of the Oct4-Sox2 relationship these studies are consistent with our findings that reduced Sox2 expression disrupts optimal Oct4 DNA-binding.

We found that reduced or complete depletion of Sox2 is associated with differentiation into extraembryonic lineages, as well as the 3 germ layers. This indicates that Sox2 plays a role in reducing the potency of nPSCs such that they cannot differentiate into extraembryonic lineages. The mechanism by which reduced Sox2 expression levels releases an inhibition on extraembryonic differentiation is likely linked to the impaired Oct4 genomic occupancy. Similar to Sox2 deletion, Oct4 deletion in ESCs causes differentiation into trophoblast-like cells (Niwa et al., 2000). However, unlike Sox2-low nPSCs, which can contribute to both embryonic and extraembryonic tissues, Oct4-low nPSCs lose the ability to differentiate into embryonic lineages (Radzisheuskaya et al., 2013). This suggests that in contrast to Oct4, Sox2 is not a key factor regulating differentiation toward embryonic lineages.

The importance of Sox2 in inhibiting extraembryonic differentiation is counter to the evidence suggesting Sox2 has a key role in extraembryonic development. Sox2 is required to derive trophoblast stem cells and is expressed in extraembryonic tissues post-implantation (Avilion et al., 2003). However, this is likely to be an *in vitro* only requirement as *Sox2*^*−/−*^ extraembryonic tissues are sufficient for development at least up to E12.5 (Avilion et al., 2003).

Morula aggregations using the Sox2-low iPSCs showed that only one out of 28 chimeric embryos exhibited trophectoderm contribution by the blastocyst stage. This is in stark contrast to E6.5 at which point 90% of all chimeric embryos display trophectoderm contribution. Interestingly, we observed cells within the epiblast upregulating the extraembryonic marker AP-2γ despite being also Oct4 positive. This is in agreement with Sox2-low iPSCs having initially a pluripotent identity, but upon differentiation showing additional competency to upregulate TE lineage markers and to contribute toward this lineage. Thus, their ability to undergo an extraembryonic lineage fate occurs after naive pluripotent cell identity exit and simultaneously with the embryonic lineage fate. The already reduced Oct4 genomic occupancy combined with the onset of the downregulation of naive factors upon initiation of cell differentiation may somehow create a window of opportunity for differentiating pluripotent cells to also acquire a TE fate.

Recently, expanded potential stem cells (EPSCs) that can contribute to both embryonic and extraembryonic lineages have been generated by two independent laboratories, by culturing ESCs in the presence of small molecules (Yang et al., 2017a, 2017b). The two laboratories used different chemical cocktails to generate the EPSCs but both resulted in cells with similar properties, suggesting a possible common mechanism. Among the added chemicals are minocycline hydrochloride (Yang et al., 2017b), a Parp1 inhibitor, and XAV939 (Yang et al., 2017a), a tankyrase inhibitor. The contributory mechanism of XAV939 to the extension of pluripotency is unclear, but it likely inhibits Parp family members TNKS1/2 and/or stabilizes AXIN (Yang et al., 2017a). Furthermore, Parp1^−/−^ ESCs have a propensity to differentiate into trophoblast (Hemberger et al., 2003). Parp1 is thought to aid Sox2 binding in ESCs (Liu and Kraus, 2017). Therefore, the previously published EPSC culture condition may reduce PARP activity and consequently reduce Sox2 DNA-binding, thus resulting in a similar phenotype to our Sox2-low nPSCs.

In conclusion, our study identifies an unexpected role of a naive pluripotency factor as a restrictor of developmental potency and provides a conciliatory mechanistic explanation for EPSCs, which also exhibit both embryonic and extraembryonic differentiation capacity. It will now be also interesting to investigate if a similar mechanism underlies recently described derivation of trophoblast cell derivatives directly from human nPSCs (Dong et al., 2020).

### Limitations of the study

A caveat of our study concerns the lack of demonstration that generated trophoblast-like cells give rise to mature and functional trophoblast cell derivatives. We find the genotype of our cells not suitable to address this question as our cells express reduced levels of Sox2. This may cause multiple embryo phenotypes, as Sox2 is normally expressed in multiple tissues, including extraembryonic ones, which are likely to preclude long-term embryo survival. In addition, our work did not intend to create cells with extra potency but rather provide an explanation on how this may arise within a naive pluripotent stem identity.

### Resource availability

#### Lead contact

Further information and requests should be directed to and will be fulfilled by the corresponding author, José C. R. Silva (jose_silva@grmh-gdl.cn).

#### Materials availability

Please contact us if you would like to request any materials.

#### Data and code availability

The single-cell RNA-sequencing data generated during this study is available in the ArrayExpress repository under accession E-MTAB-9931.

## Methods

All methods can be found in the accompanying Transparent methods supplemental file.

## Supplemental information

Document S1. Transparent methods and Figures S1–S4

Data S1. List of differentially expressed genes between Sox2-low iPSCs and control iPSCs, related to Figure 6
