# Supplementary material for: Sox2 modulation increases naïve pluripotency plasticity
Source: iScience. 2021 Feb 6;24(3):102153. doi: 10.1016/j.isci.2021.102153 (PMC7903329; doi:10.1016/j.isci.2021.102153)
Supplement: Document S1. Transparent methods and Figures S1–S4 [file mmc1.pdf]

iScience, Volume *24*

Supplemental Information

Sox2 modulation increases naïve pluripotency plasticity

Kathryn C. Tremble, Giuliano G. Stirparo, Lawrence E. Bates, Katsiaryna Maskalenka, Hannah T. Stuart, Kenneth Jones, Amanda Andersson-Rolf, Aliaksandra Radzisheuskaya, Bon-Kyoung Koo, Paul Bertone, and José C.R. Silva

Transparent methods

Plasmids
pMXs c-Myc, pMXs Oct4, pMXs Sox2, pMXs Klf4 and pSpCas9(BB)-2A-GFP (Addgene). pPyCAG-MST-IRES-Puro, pPyCAG-eGFP-IRES-Zeo (Austin Smith). pPB-CAG-DEST-hygro, pCAG-CreERT2NLS-IRES-bsd (Joerg Betschinger). PB-CAG-Sox2-hygro was used for reprogramming and for the rescue of Sox2-low iPSCs.

Cell culture
PLAT-E cells and (where stated) iPSCs were cultured in GMEM basal medium (GMEM (Sigma-Aldrich), 1xNEAA (Gibco), 1xpenicillin-streptomycin (Sigma-Aldrich), 1mM sodium pyruvate (Sigma-Aldrich), 0.1mM 2-mercaptoethanol (ThermoFisher Scientific) and 2mM L-glutamine (ThermoFisher Scientific)) with 10% FCS (Labtech) and 20ng/ml mouse LIF (University of Cambridge). NSCs were cultured in DMEM/F12 (ThermoFisher Scientific), 1xNEAA, 0.1mM 2-mercaptoethanol, 1xpenicillin-streptomycin, 1:100 v/v B27 supplement (ThermoFisher Scientific), 1:200 v/v N2 supplement (University of Cambridge), 4.5µM HEPES (ThermoFisher Scientific), 0.03M glucose and 120µg/ml BSA (ThermoFisher Scientific) supplemented with 10ng/ml EGF (Peprotech) and 20ng/ml FGF2 (University of Cambridge). iPSCs were cultured in KSR basal medium (GMEM basal medium with 10% KSR, 1% FCS) or N2B27 basal medium (DMEM/F12 and Neurobasal (ThermoFisher Scientific) in a 1:1 ratio, 1xpenicillin-streptomycin, 0.1mM 2-mercaptoethanol, 2mM L-glutamine, 1:200 v/v N2 and 1:100 v/v B27 supplement) with additional 20ng/ml mouse LIF, CHIR99021 3µM and PD0325901 1µM (both Biotechnology Center TU Dresden, Stewart lab). Additional chemicals used: hygromycin B 200 µg/ml (ThermoFisher Scientific), blasticidin 40 µg/ml (ThermoFisher Scientific), puromycin 1µg/ml (ThermoFisher Scientific), zeocin 100µg/ml (ThermoFisher Scientific), 4-hydroxytamoxifen 500 nM.

Reprogramming
Separate PLAT-E cultures were transfected with 9µg of each pMX plasmid using FuGENE 6 (Roche) for retroviral production. 48 hours later the supernatants were collected, combined, and mixed with 4µg/ml Polybrene (Sigma-Aldrich) and filtered through a 0.45 µm cellulose acetate filter. This was applied to NS cells for 24 hours, after which they were returned to NS medium for 2 days, before being placed in serum/LIF to form reprogramming intermediates (preiPSCs). These were then placed in KSR2iLIF to form iPSCs.

Cell differentiation
For embryoid body assays, $1.5x10^6$ cells were plated in suspension into serum basal media without LIF in 90mm low-attachment dishes for 7 days with media changed every other day.

Neural differentiation
$1x10^5$ cells were plated directly into 6-well plates, previously coated with laminin for 2 hrs at 37 degrees, containing N2B27 plus 1µM Alk inhibitor (A83-01) differentiation medium and then cultured in a low oxygen incubator. Medium was changed every day.

Western blotting
The primary antibodies used were: rat monoclonal against Sox2 (1:2000, eBioscience, 149811); mouse monoclonal against α-tubulin (1:10000, Abcam, ab7291). The secondary antibodies were HRP-linked antibodies against rat or mouse IgG (GE Healthcare).

Embryo staining
Embryos were fixed with 4% paraformaldehyde and mounted in vectashield antifade mounting medium (Vector Laboratories). Blastocysts were permeabilised using 100% methanol and blocked using 2% donkey serum, 0.1% BSA and 0.2% Triton X-100. Post-implantation embryos were permeabilised in 0.25% Triton X-100/PBS and blocked using 0.01% Triton X-100/PBS and 3% donkey serum. Primary antibodies used were goat polyclonal against Sox17 (1:500, R and D systems, AF1924), mouse monoclonal against Cdx2 (1:500, Biogenex, MU392A-UC), AP-2γ (1:100, Cell Signalling Technology, 2320), rat monoclonal against GFP (1:200, Nacalai, 04404-26), mouse monoclonal against Oct4 (1:100, Santa Cruz, sc-5279). Secondary antibodies were Alexa Fluor antibodies (ThermoFisher Scientific) and were used at a concentration of 1:500.

Morula aggregation

E2.5 CD1 embryos were combined with iPSCs and transferred to recipient mice to assess the contribution in post-implantation development or were cultured *in vitro* for 2 days to assess blastocyst contribution.

Cell transfection
Nucleofection (NSCs) was performed using the AMAXA Nucleofection Technology (Lonza, VCA-1003). Lipofection (ESCs, iPSCs) was performed using Lipofectamine 2000 Transfection Reagent (ThermoFisher Scientific).

Western Blot Quantification
Quantification was performed using the 'Gels' tool in ImageJ Fiji. Background-subtracted relative intensity (BSRI) was calculated for each band of interest. Protein of interest (Sox2) BSRI values were then normalized to the corresponding loading control (tubulin) BSRI values and ESCs.

Derivation of Rex1-GFP Neural Stem cells
Rex1$^{dGFP.IRES.bsd/dGFP.IRES.bsd}$ homozygous 129 studs (Kalkan et al., 2017) were crossed with wild-type 129 females and heterozygous *Rex1*$^{+/dGFP.IRES.bsd}$ Neural Stem cells (referred to as Rex1-GFP reporter) were derived from resultant E13.5 embryos as previously described (Pollard et al., 2006). Rex1-GFP Neural Stem cells have a 129 strain genetic background.

Generation of CRISPR/Cas9 cell lines
Sox2$^{-/-}$ NSCs were generated by nucleofecting NSCs with 4µg CRISPR/gRNA plasmid and single-cell sorting 48 hours later to generate clonal cell lines. The generation of Sox2 FLIP/FLIP ESCs has been previously described (Andersson-Rolf et al., 2017). Sox2 FLIP/FLIP ESCs have a 129 strain genetic background.

Chromatin Immunoprecipitation (ChIP)
ChIP was performed as previously described (Radzisheuskaya et al., 2013) with minor changes. Briefly, $1x10^7$ cells were fixed for 10 min in 0.4% formaldehyde then washed with ice-cold PBS. Cells were incubated in lysis buffer 1 (50 mM HEPES pH 7.5, 140 mM NaCl, 1 mM EDTA, 10% Glycerol, 0.5% NP40, 0.25% Tx100) then in lysis buffer 2 (10 mM Tris pH 8.0, 200 mM NaCl, 1 mM EDTA, 0.5 mM EGTA) for 10 min each. Nuclei were resuspended in shearing buffer (1% SDS, 10 mM EDTA, 50 mM Tris pH 8.0) and sonicated to an average fragment size of 300-500 bp. Chromatin was diluted 1:10 in dilution buffer (50 mM Tris pH 8.0, 167 mM NaCl, 1.1% Tx100, 0.11% Na-deoxycholate) and cleared with isotype IgG coated protein G Dynabeads (ThermoFisher Scientific). A portion of chromatin was taken as input control. Chromatin was then incubated overnight at 4°C with 1.5µg Rabbit anti-Oct4 antibody (Abcam; ab19857) antibody or 2µg Rabbit normal IgG (Santa Cruz Biotechnology; sc-2027). Chromatin-antibody mix was incubated with pre-blocked protein G dynabeads for 1 hour at 4°C. These were then washed twice in low salt wash buffer (50 mM Tris pH 8.0, 0.1% SDS, 0.1% Na-deoxycholate, 1% Tx100, 150 mM NaCl, 1 mM EDTA, 0.5 mM EGTA), once in high salt wash buffer (50 mM Tris pH 8.0, 0.1% SDS, 0.1% Na-deoxycholate, 1% Tx100, 500 mM NaCl, 1 mM EDTA, 0.5 mM EGTA), once in LiCl wash buffer (50 mM Tris pH 8.0, 250 mM LiCl, 0.5% Na-deoxycholate, 0.5% NP40, 1 mM EDTA, 0.5 mM EGTA) and twice in TE wash buffer (50 mM Tris pH 8.0, 10 mM EDTA, 5 mM EGTA). Bound chromatin was then eluted at 65°C in elution buffer (1% SDS, 0.1 M $NaHCO_3$). Crosslinking was reversed through overnight incubation at 65°C for samples and inputs. DNA was purified using the QIAquick PCR Purification Kit (Qiagen) according to the manufacturer's protocol. DNA was quantitated by SYBR Green (ThermoFisher Scientific) qPCR, with a standard curve to ensure linear amplification. Immunoprecipitation efficiency was calculated relative to input = 1.

Primers for ChIP

Primer	Sequence
Nanog proximal enhancer forward	GCAGCCGTGGTTAAAAGATG
Nanog proximal enhancer reverse	GAAGCTGTAAGGTGACCCAGA
Oct4 distal enhancer forward	GCATAACAAAGGTGCATGATAGCT
Oct4 distal enhancer reverse	AAATAAAGGCAGCGACTTGGAA
Klf2 proximal enhancer forward	CTGCACAAAGGGCTTAGAGG
Klf2 proximal enhancer reverse	CCTCATTTGCACCACACCTA

RNA extraction, cDNA synthesis and qPCR

Total RNA was isolated using the RNeasy Mini kit (QIAGEN). 1µg RNA was reverse-transcribed using SuperScript III First-Strand Synthesis SuperMix for qRT-PCR (ThermoFisher Scientific). The resultant cDNA was analysed by quantitative PCR using TaqMan Fast Universal PCR Master Mix (ThermoFisher Scientific) with TaqMan Gene Expression Assays and/or KiCqStart assays, or with Fast SYBR Green Master Mix (ThermoFisher Scientific) using primers. qRT-PCR experiments were performed in triplicate on a StepOnePlus Real-Time PCR System (Applied Biosystems). Delta Ct values were normalised to GAPDH and raised to the power of -2. Standard deviations refer to technical replicates.

**Primers used with SYBR Green**	
GAPDH FP	CCCACTAACATCAAATGGGG
GAPDH RP	CCTTCCACAATGCCAAAGTT
Olig2 FP	CTGCTGGCGCGAAACTACAT
Olig2 RP	CGCTCACCAGTCGCTTCAT
BLBP FP	AGACCCGAGTTCCTCCAGTT
BLBP RP	ATCACCACTTTGCCACCTTC
FoxA1 FP	ATGAGAGCAACGACTGGAACA
FoxA1 RP	TCATGGAGTTCATAGAGCCCA
Pl-1 FP	ATTTTGACTACCCTGCTTGGTCT
Pl-1 RP	TCTACATAACTGAGGAGGGGAAAG
Hand1 FP	CCCCTCTTCCGTCCTCTTAC
Hand1 RP	CTGCGAGTGGTCACACTGAT
Eomes FP	CCTGGTGGTGTTTTGTTGTG
Eomes RP	TTTAATAGCACCGGGCACTC
**ThermoFisher custom Taqman probes**	
Endogenous Oct4 FP	TTCCACCAGGCCCCC
Endogenous Oct4 RP	GGTGAGAAGGCGAAGTCTGAAG
Endogenous Oct4 probe	FAM-CCCACCTTCCCCATGGCT-MGB
Retroviral Sox2 FP	TGGTACGGGAAATCACAAGTTTGTA
Retroviral Sox2 RP	GCCCGGCGGCTTCA
Retroviral Sox2 probe	FAM-CTCCGTCTCCATCATGTTAT-MGB
Retroviral cMyc FP	TGGTACGGGAAATCACAAGTTTGTA
Retroviral cMyc RP	GGTCATAGTTCCTGTTGGTGAAGTT
Retroviral cMyc probe	FAM-CCCTTCACCATGCCCC-MGB
Retroviral Klf4 FP	TGGTACGGGAAATCACAAGTTTGTA
Retroviral Klf4 RP	GAGCAGAGCGTCGCTGA
Retroviral Klf4 probe	FAM-CCCCTTCACCATGGCTG-MGB
Retroviral Oct4 FP	TGGTACGGGAAATCACAAGTTTGTA
Retroviral Oct4 RP	GGTGAGAAGGCGAAGTCTGAAG
Retroviral Oct4 probe	FAM-CACCTTCCCCATGGCTG-MGB
**Sigma-Aldrich KiCqStart Probes**	
Krt7	MMUS_NM_033073_1
**ThermoFisher Taqman Probes**	
Total Oct4	Mm00658129_gH
GAPDH	4352339E
Nanog	Mm02384862_g1
Esrrb	Mm00442411_m1
FGF5	Mm00438918_m1
T Brachyury	Mm01318252_m1
Gata4	Mm00484689_m1
Klf2	Mm01244979_g1
Sox1	Mm00486299_s1

Ascl1	Mm03058063_m1
Pax6	Mm00443081_m1
Zeb2	Mm00497193_m1
Elf5	Mm00468732_m1

ScRNA-seq library preparation
Single cells were index-sorted individually by FACS (BD Influx 5) into wells of a 96-well PCR plate containing lysis buffer. ScRNA-seq was performed as previously described (Nestorowa et al., 2016; Picelli et al., 2014; Wilson et al., 2015). The Illumina Nextera XT DNA kit was used to prepare libraries. Pooled libraries were sequenced on the Illumina HiSeq 4000 (single-end 125bp reads).

RNA-seq data analysis
Sequencing reads were aligned to mouse genome reference GRCm38/mm10 with STAR (Dobin et al., 2013) using the two-pass method for novel splice detection (Engström et al., 2013). GENCODE M12 mouse gene annotation from Ensembl release 87 (Yates et al., 2016) was used for read alignment and splice junction donor/acceptor overlap settings were tailored to the read length of each dataset. Alignments to gene loci were quantified with HTSeq-count (Anders et al., 2015) based on annotation from Ensembl release 87. Quality control was performed according to (Stirparo et al., 2018). Briefly, sequencing libraries with fewer than 500,000 mapped reads were excluded from subsequent analyses. Read distribution bias across gene bodies was computed as the ratio between the total reads spanning the 50th to the 100th percentile of gene length, and those between the first and 49th. Samples with ratio >2 were not considered further. Stage-specific outliers were screened by principal component analysis.

Published scRNA-seq datasets
Sequencing data corresponding to single-cell mouse embryo profiling studies SRP110669 (Mohammed et al., 2017) (E3.5, E4.5), SRP020490 (Deng et al., 2014) (trophectoderm cells) and E-MTAB-7901 (Stuart et al., 2019) (ESCs) were obtained from the European Nucleotide Archive (Toribio et al., 2017) and from ArrayExpress repository and processed as above.

Transcriptome analysis
Principal component and cluster analyses were performed based on log2 FPKM values and were computed with FactoMineR (Lê et al., 2008) in addition to custom scripts. Default parameters were used unless otherwise indicated. For global analyses, genes that registered zero counts in all single-cell samples were omitted. Euclidean distance and complete linkage were used for cluster analyses unless otherwise indicated. Differential expression analysis was performed with scda (Kharchenko et al., 2014), that fits individual error models for assessment of differential expression between groups of cells. DAVID Bioinformatics Resources 6.7 (Huang et al., 2009) was used for computing the enriched biological processes, using as input list the modulated genes (with padj value < 0.05) between mutant cells and wt/rescue cells. Genes exhibiting the greatest expression variability (and thus contributing substantial discriminatory power) were identified by fitting a non-linear regression curve between average log2 FPKM and the square of coefficient of variation. Indicated specific thresholds were applied along the x-axis (average log2 FPKM) and y-axis (CV2) to identify the most variable genes. Cumulative sum was computed by performing the sequential sums of log2 expression values for genes expressed in TE or ICM stage.

Data and Code Availability

The single-cell RNA sequencing data generated during this study is available in the ArrayExpress repository under accession E-MTAB-9931

Supplemental References

Anders, S., Pyl, P.T., and Huber, W. (2015). HTSeq-A Python framework to work with high-throughput sequencing data. Bioinformatics *31*, 166–169.

Andersson-Rolf, A., Mustata, R.C., Merenda, A., Kim, J., Perera, S., Grego, T., Andrews, K., Tremble, K., Silva, J.C.R., Fink, J., et al. (2017). One-step generation of conditional and reversible gene knockouts. Nat Methods.

Deng, Q., Ramsköld, D., Reinius, B., and Sandberg, R. (2014). Single-cell RNA-seq reveals dynamic, random monoallelic gene expression in mammalian cells. Science (80-) *343*, 193–196.

Dobin, A., Davis, C.A., Schlesinger, F., Drenkow, J., Zaleski, C., Jha, S., Batut, P., Chaisson, M., and Gingeras, T.R. (2013). STAR: Ultrafast universal RNA-seq aligner. Bioinformatics.

Engström, P.G., Steijger, T., Sipos, B., Grant, G.R., Kahles, A., Rätsch, G., Goldman, N., Hubbard, T.J., Harrow, J., Guigó, R., et al. (2013). Systematic evaluation of spliced alignment programs for RNA-seq data The RGASP Consortium Europe PMC Funders Group. Nat Methods.

Huang, D.W., Sherman, B.T., and Lempicki, R.A. (2009). Systematic and integrative analysis of large gene lists using DAVID bioinformatics resources. Nat Protoc.

Kalkan, T., Olova, N., Roode, M., Mulas, C., Lee, H.J., Nett, I., Marks, H., Walker, R., Stunnenberg, H.G., Lilley, K.S., et al. (2017). Tracking the embryonic stem cell transition from ground state pluripotency. Development *144*, 1221–1234.

Kharchenko, P. V., Silberstein, L., and Scadden, D.T. (2014). Bayesian approach to single-cell differential expression analysis. Nat Methods *11*, 740–742.

Lê, S., Josse, J., and Husson, F. (2008). FactoMineR : An R Package for Multivariate Analysis. J Stat Softw *25*, 253–258.

Mohammed, H., Hernando-Herraez, I., Savino, A., Scialdone, A., Macaulay, I., Mulas, C., Chandra, T., Voet, T., Dean, W., Nichols, J., et al. (2017). Single-Cell Landscape of Transcriptional Heterogeneity and Cell Fate Decisions during Mouse Early Gastrulation. Cell Rep *20*, 1215–1228.

Nestorowa, S., Hamey, F.K., Pijuan Sala, B., Diamanti, E., Shepherd, M., Laurenti, E., Wilson, N.K., Kent, D.G., and Göttgens, B. (2016). A single cell resolution map of mouse haematopoietic stem and progenitor cell differentiation. Blood blood-2016-05-716480.

Picelli, S., Faridani, O.R., Björklund, Å.K., Winberg, G., Sagasser, S., and Sandberg, R. (2014). Full-length RNA-seq from single cells using Smart-seq2. Nat Protoc *9*, 171–181.

Pollard, S.M., Conti, L., Sun, Y., Goffredo, D., and Smith, A. (2006). Adherent neural stem (NS) cells from fetal and adult forebrain. Cereb Cortex *16*.

Radzisheuskaya, A., Chia Gle, B., dos Santos, R.L., Theunissen, T.W., Castro, L.F.C., Nichols, J., Silva, J.C.R., Chia, G.L. Bin, dos Santos, R.L., Theunissen, T.W., et al. (2013). A defined Oct4 level governs cell state transitions of pluripotency entry and differentiation into all embryonic lineages. Nat Cell Biol *15*, 579–590.

Stirparo, G.G., Boroviak, T., Guo, G., Nichols, J., Smith, A., and Bertone, P. (2018). Integrated analysis of single-cell embryo data yields a unified transcriptome signature for the human pre-implantation epiblast. Dev.

Stuart, H.T., Stirparo, G.G., Lohoff, T., Bates, L.E., Kinoshita, M., Lim, C.Y., Sousa, E.J., Maskalenka, K., Radzisheuskaya, A., Malcolm, A.A., et al. (2019). Distinct Molecular Trajectories Converge to Induce Naive Pluripotency. Cell Stem Cell *25*, 388-406.e8.

Wilson, N.K., Kent, D.G., Buettner, F., Shehata, M., Macaulay, I.C., Calero-Nieto, F.J., Sánchez Castillo, M., Oedekoven, C.A., Diamanti, E., Schulte, R., et al. (2015). Combined Single-Cell Functional and Gene Expression Analysis Resolves Heterogeneity within Stem Cell Populations. Cell Stem Cell *16*, 712–724.

Yates, A., Akanni, W., Amode, M.R., Barrell, D., Billis, K., Carvalho-Silva, D., Cummins, C., Clapham, P., Fitzgerald, S., Gil, L., et al. (2016). Ensembl 2016. Nucleic Acids Res *44*, D710–D716.

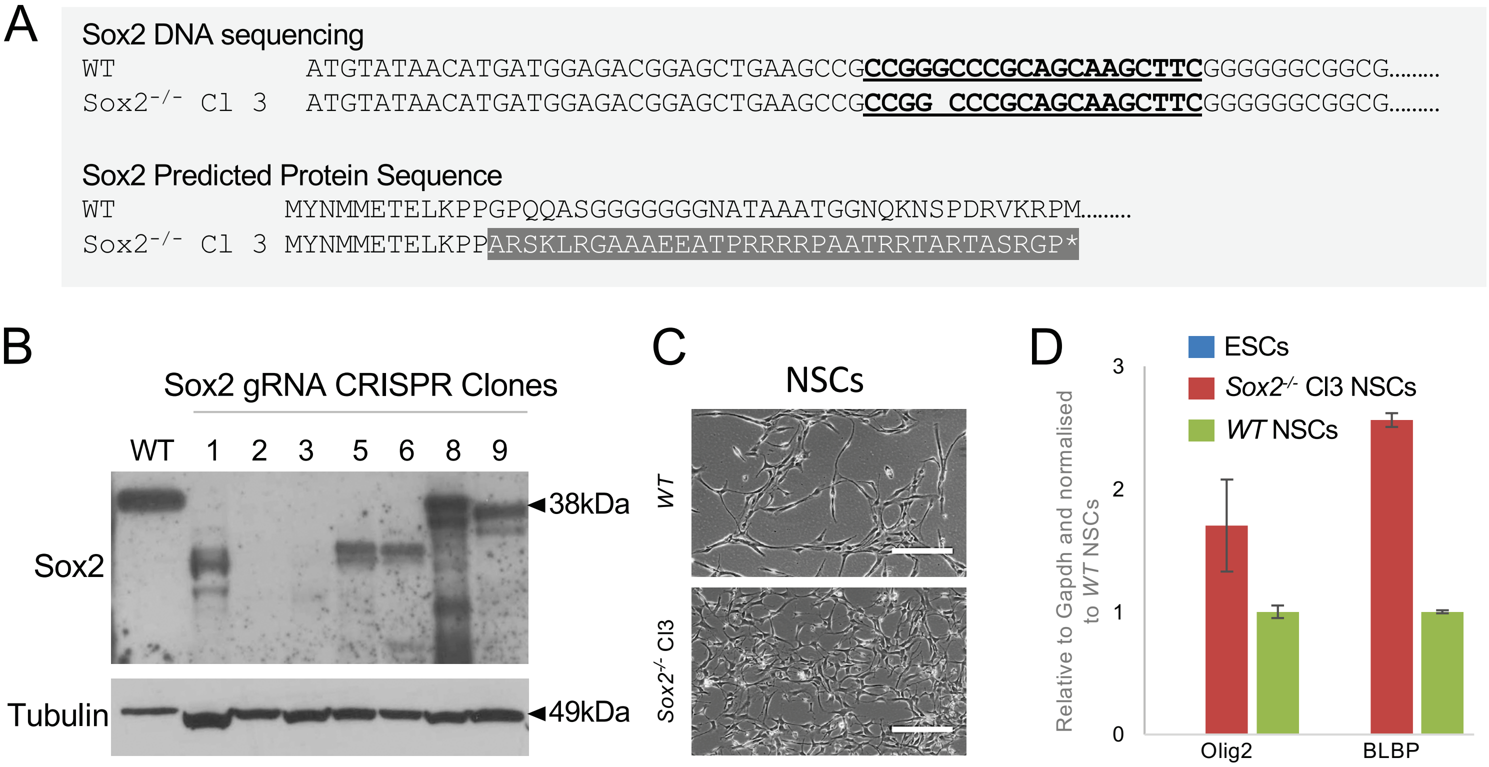

Figure S1- Generation of *Sox2$^{-/-}$* NSCs. Related to Figure 1.

A) Sequence of start of coding region of *Sox2* amplified by genomic PCR for *Sox2$^{-/-}$* neural stem cell (NSC) clone 3 with Cas9 targeting gRNA binding site underlined aligned with the WT Sox2 coding region to show the mutation (deletion of 1 guanine). The predicted protein sequence from the *Sox2$^{-/-}$* NSC clone 3 is shown aligned with WT sequence, with the sequence diversion highlighted. * = stop codon.

B) Western blot for Sox2 (≈40kDa) and Tubulin (≈50kDa) protein expression in WT and clonal NSC lines after transfection with Sox2 gRNA/Cas9.

C) Phase images of *WT* and *Sox2$^{-/-}$* clone (Cl) 3 NSCs.

D) qRT-PCR analysis of neural markers (Olig2 and BLBP) in *WT* and *Sox2$^{-/-}$* Cl3 NSCs and ESCs. Error bars indicate standard deviation of replicate qPCR reactions (n=3).

Scale bars = 200µm.

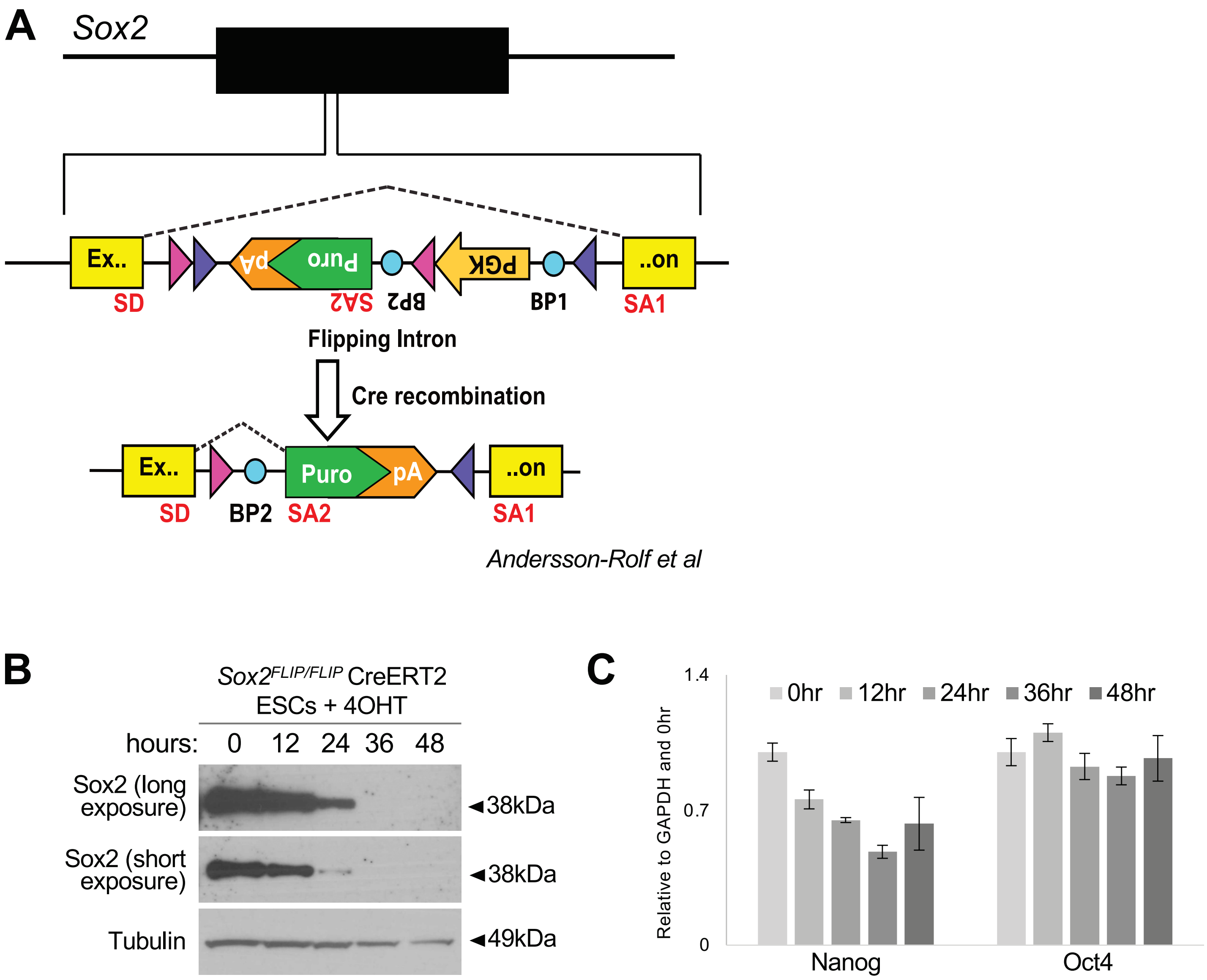

Figure S2- *Sox2*$^{FLIP/FLIP}$ ESCs experimental optimisation. Related to Figure 3.

A) Schematic of Sox2$^{FLIP/FLIP}$ ESCs. Please note that after Cre mediated recombination Sox2 protein becomes truncated. SD indicates splice donor site. BP1 and SA1, and BP2 and SA2 indicate pairs of branch points and splice acceptor sites that are used before and after Cre recombination respectively. Pink and purple triangles indicate LoxP variants (Lox5171 and LoxP1 respectively). Dashed line indicates splice site usage.

B) Western blot of 48 hour timecourse of tamoxifen (4OHT) treatment of Sox2$^{FLIP/FLIP}$ CreERT2 ESCs.

C) qRT-PCR of pluripotency factor expression during tamoxifen timecourse. Error bars indicate standard deviation of replicate qPCR reactions (n=3).

A

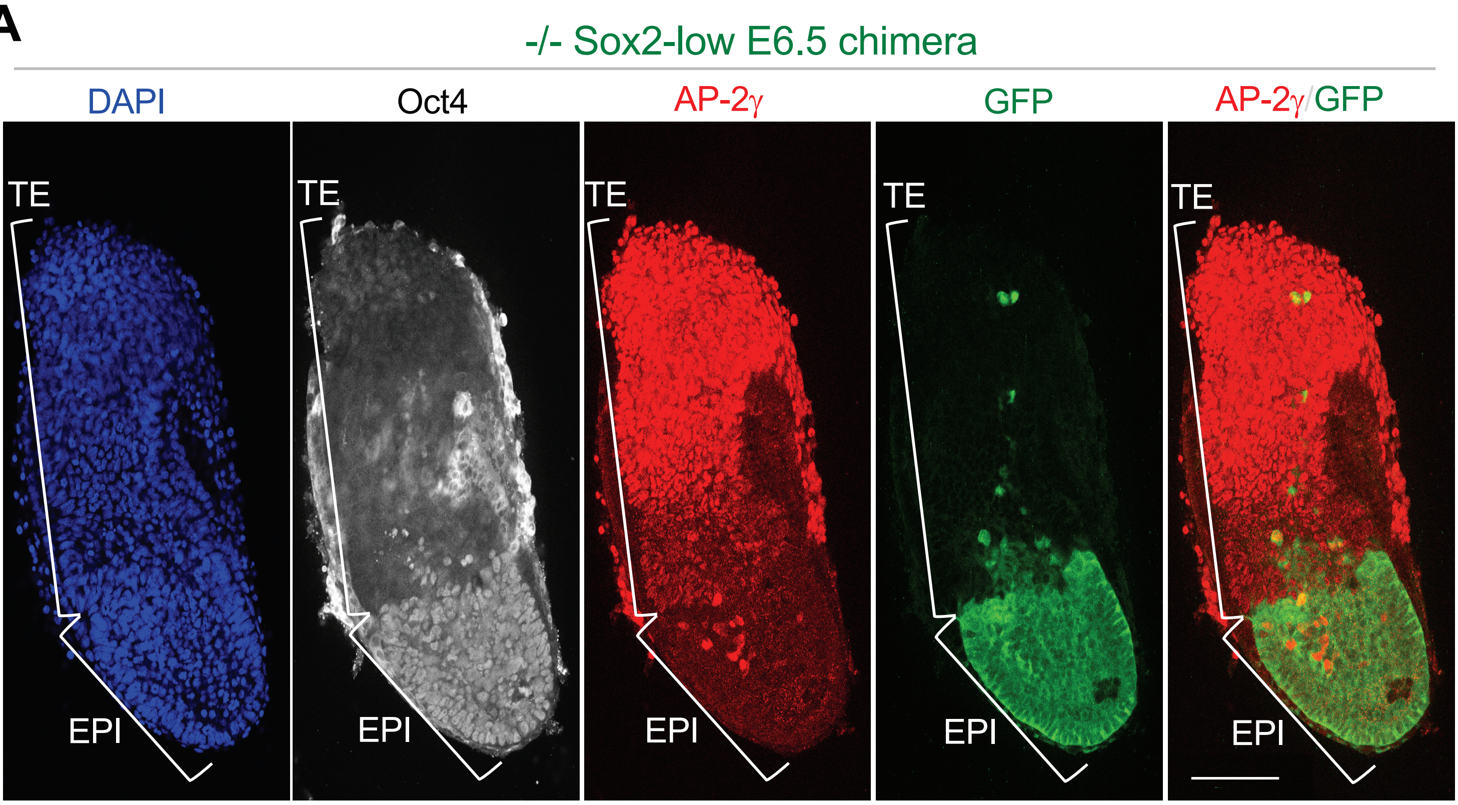

B

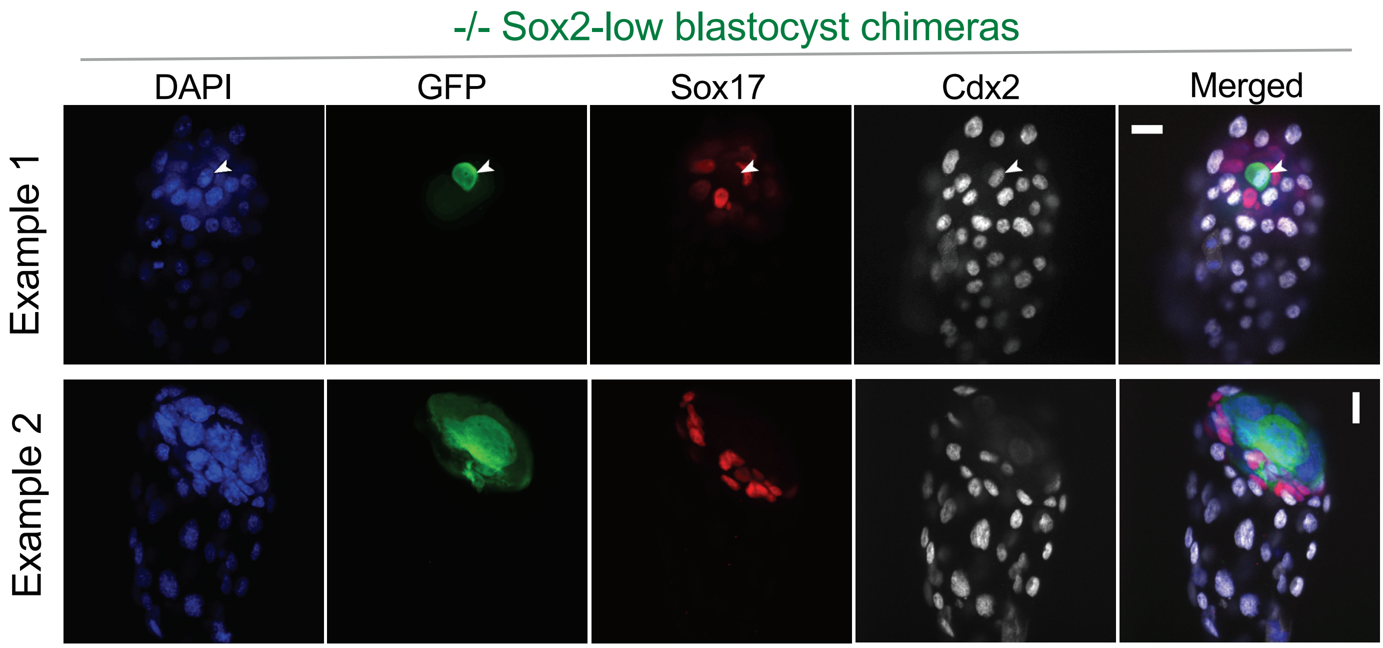

C

-/- Sox2-low blastocyst chimeras

Epiblast only	27
Trophectoderm (cdx2 positive)	1

Figure S3 - Sox2-low iPSCs exhibit increased plasticity in vivo. Related to Figure 5.
A) Single confocal microscopy section of *Sox2*$^{-/-}$ (-/-) Sox2-low iPSCs (GFP) E6.5 chimeric embryos stained with trophoblast (AP-2**γ**) and epiblast (Oct4) markers. Epiblast (EPI) and Trophoblast/trophectoderm (TE) embryo domains are indicated. Scale bars = 100**µ**m.
B) Immunofluorescence staining with Cdx2 (trophectoderm) and Sox17 (hypoblast) of cultured embryos after morula injection with *Sox2*$^{-/-}$ (-/-) Sox2-low iPSCs expressing constitutively a GFP transgene. Scale bar = 20**µ**m.
C) Table showing compartmental contribution of -/- Sox2-low iPSCs.

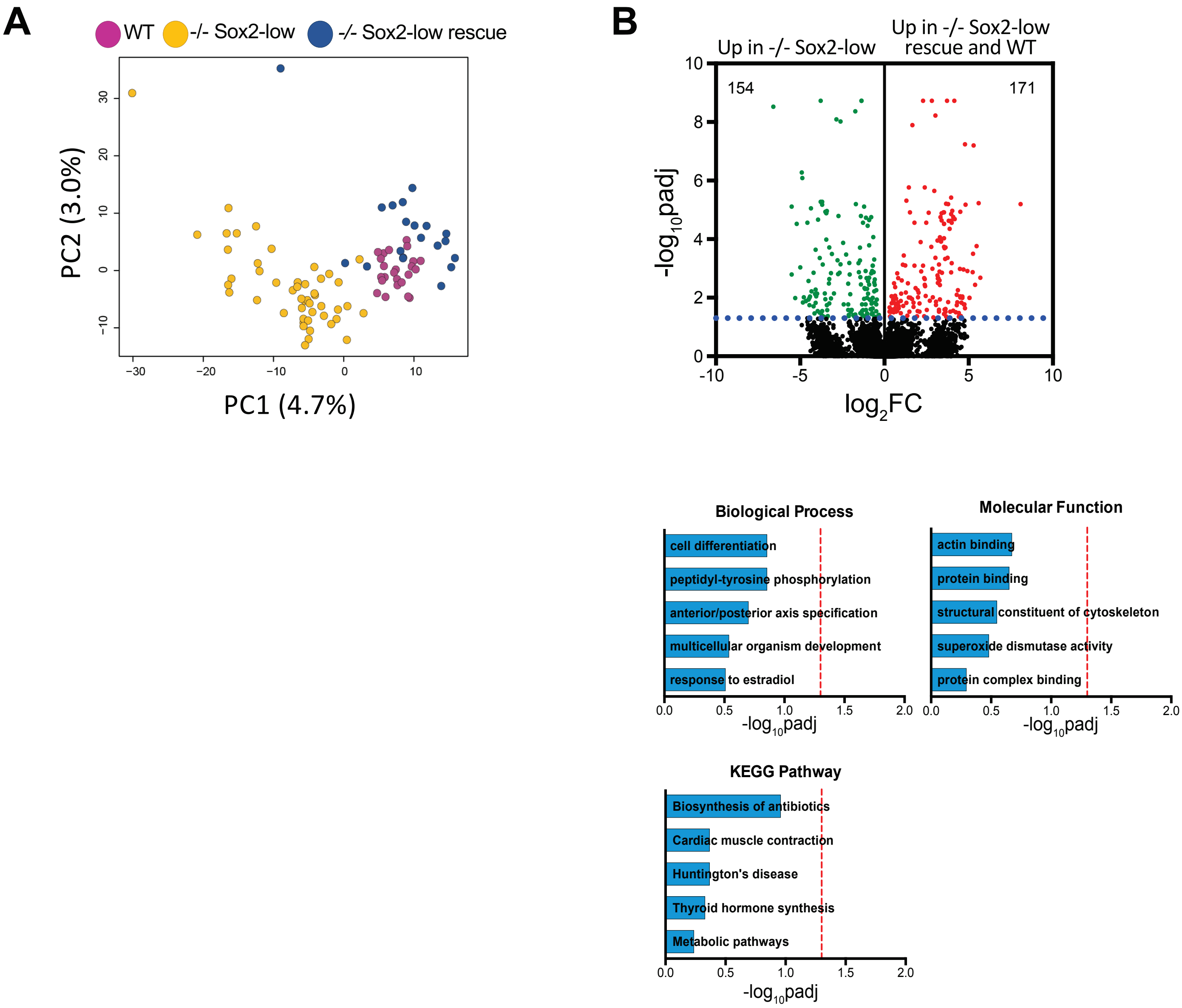

Figure S4- Sox2-low iPSCs do not display gene ontology differences to control cell lines. Related to Figure 6
A) PCA plot computed with the top variable genes for *Sox2*$^{-/-}$ (-/-) Sox2-low and control iPSCs, which include WT and -/- Sox2-low rescued with a constitutive Sox2 transgene (-/- Sox2-low rescue) (FPKM > 1, logCV2 >0.5, n=1951). The cells are of same genetic background, even the same cell line (parental, knock-out and rescue). They have also been done on same plate for library prep and sequenced in the same lane meaning that the variance is not technical batch effect of the sequencing.
B) Volcano plot and gene ontology enrichment for genes differentially expressed between -/-Sox2-low and control iPSCs (combined WT and -/- Sox2-low rescue iPSCs). log FC indicates comparison between -/- Sox2-low and control iPSCs. Negative value means gene is down regulated in WT and -/- Sox2-low rescue, while positive value indicates gene is upregulated in WT and -/- Sox2-low rescue compared to -/-Sox2-low iPSCs. Significance threshold was defined at $-\log_{10}$padj=1.3.
